# Deregulation of angiopoietin-like 4 slows ovarian cancer progression through vascular endothelial growth factor receptor 2 phosphorylation

**DOI:** 10.1186/s12935-021-01865-4

**Published:** 2021-03-16

**Authors:** Yuxian Wu, Jinghai Gao, Xiaojun Liu

**Affiliations:** Department of Obstetrics and Gynaecology, Changzheng Hospital, Naval Medical University, 415 Fengyang Road, Shanghai, 200003 China

**Keywords:** Angiopoietin-Like 4, Ovarian cancer, Angiogenesis, Vascular endothelial growth factor receptor 2, Vascular endothelial-cadherin

## Abstract

**Background:**

As a tissue-specific proangiogenic or antiangiogenic agent, angiopoietin-like 4 (ANGPTL4) has recently gained attention in many diseases, such as metabolic syndrome, cardiovascular disease and cancer. However, the roles of ANGPTL4 in angiogenesis and tumor growth in epithelial ovarian cancer, the most lethal gynecologic malignancy, remain unclear.

**Objective:**

To identify a novel mechanism of ANGPTL4 inhibition in epithelial ovarian cancer.

**Methods:**

Western blot, quantitative reverse transcription PCR, and immunofluorescence analyses were applied to evaluate ANGPTL4 expression in ovarian cancer cell lines. Cell proliferation, migration, and invasion were investigated through 5-ethynyl-2′-deoxyuridine (EdU) incorporation, CCK-8 and Transwell assays. The expression of epithelial-mesenchymal transition (EMT)-related proteins in ovarian cancer cells and tumor-bearing mice was evaluated. CD31 staining was used to identify tumor angiogenesis. Immunoprecipitation was performed to examine the regulatory relationship between ANGPTL4 and the vascular endothelial growth factor receptor 2 (VEGFR2)/vascular endothelial (VE)-cadherin/Src complex. VEGFR2 phosphorylation at Y949 and VE-cadherin expression were assessed by western blotting. Inactivation of VEGFR2 Y949 phosphorylation was achieved in a MISIIR-TAg *VEGFR2*^*Y949F/Y949F*^ mouse model.

**Results:**

Here, we demonstrated that ANGPTL4 was overexpressed in A2780 and CAOV3 ovarian cancer cells. In vitro assays indicated that inhibition of ANGPTL4 by lentiviral small interfering RNA does not alter ovarian cancer cell proliferation, migration, invasion, and EMT, while ANGPTL4 silencing exhibited significant inhibitory effects on tumor angiogenesis, growth, and metastasis in vivo. Immunoprecipitation analysis showed that suppression of ANGPTL4 was accompanied by dissociation of the VEGFR2/VE-cadherin/Src complex and phosphorylation of VEGFR2 Y949 in A2780 and CAOV3 ovarian tumors. Inactivation of VEGFR2 Y949 phosphorylation in MISIIR-TAg *VEGFR2*^*Y949F/Y949F*^ mice abolished all tumor-suppressive effects of ANGPTL4 inhibition in spontaneous ovarian carcinoma.

**Conclusions:**

Overall, our results indicate that ANGPLT4 silencing delays tumor progression in specific types of ovarian cancer and may be a potential target for individualized treatment of ovarian cancer.

**Supplementary Information:**

The online version contains supplementary material available at 10.1186/s12935-021-01865-4.

## Background

Epithelial ovarian cancer is the most lethal gynecologic malignancy, with a five-year survival rate of only approximately 20% and claiming the lives of nearly 150,000 women annually worldwide [1, 2]. The symptoms of ovarian cancer are nonspecific and often attributed to other more common ailments; therefore, correct diagnosis is usually made only when widespread metastasis occurs beyond the ovaries. Standard treatment includes surgical debulking in combination with chemotherapy with a taxane-containing platinum doublet [3]. Although most patients initially respond favorably to this combination therapy regimen, many patients relapse with recurrent disease that is resistant to chemotherapy; therefore, novel targeted drugs are still urgently needed.

Tumor endothelial cells that form the tumor vasculature play a conventional role in nutrient transport to support tumor proliferation and facilitate cancer metastasis. The hypoxic tumor microenvironment and the accompanying inflammation are attributed to the poor condition of the tumor vasculature, which leads to the production of various growth regulatory factors, including vascular endothelial growth factors (VEGFs) [4] and angiopoietin-like proteins (ANGPTLs) [5]. VEGFs promote angiogenesis and induce increased vascular leakage by destabilizing adherens junctions established by homophilic interactions between vascular endothelial (VE)-cadherin proteins expressed on endothelial cells [6]. ANGPTLs, which are considered orphan ligands, share functional properties with VEGFs [7, 8]. Angiopoietin-like protein 4 (ANGPTL4) is a glycosylated adipokine that belongs to the ANGPTL family, and it acts as a tissue-specific regulator of vascular permeability [9–11] that modulates the survival and adhesion of endothelial cells in vitro [12, 13] and vascular angiogenesis in vivo [9, 14]. In addition, the transforming growth factor-beta 2 (TGF-β2)/ANGPTL4 axis was reported to be responsible for breast cancer brain metastasis in a mouse model [15]. Previous studies reported that high ANGPTL4 expression was detected in ovarian cancer xenograft mouse models and patients and was associated with shorter relapse-free survival times in serous ovarian carcinoma [16, 17]. However, whether ANGPTL4 deletion has clinical benefit in ovarian cancer therapy is unknown, and the related molecular mechanisms are obscure.

Two endothelial cell receptors, the VEGF receptor 1 (VEGFR1) and VEGFR2 tyrosine kinases, are responsible for the roles of VEGFs in vascular growth modulation [18]. VEGFs are bound and neutralized by VEGFR1, exerting negative regulatory effects on endothelial cells, while VEGFR2 is vital in all known aspects of VEGF biology [18]. Phosphorylation of several tyrosine phosphosites (Y949, Y1052, 1057, Y1173, and Y1212; the numbers correspond to the mouse sequence) activates VEGFR2 [19]. In the normal vasculature, VEGFR2, VE-cadherin, and Src form a complex to maintain vascular integrity [20]. Interestingly, phosphorylation of VEGFR2 at the Y949 residue (pY949) in mice (pY951 in humans) enables Src phosphorylation at Y416 and subsequently dissociates the VEGFR2/VE-cadherin/Src complex at endothelial junctions to decrease tumor vascularization and growth and increase vascular permeability [19, 21]. These results prompted us to investigate the tumor-suppressive mechanisms of ANGPTL4 by analogy with the VEGFR2 pY949/VE-cadherin/Src pathway.

The aims of the current study were to determine ANGPTL4 expression in ovarian cancer cell lines and the effects of ANGPTL4 knockdown by RNA interference (RNAi) on cancer cell lines and tumors in vivo and in vitro. Additionally, the mechanisms of ANGPTL4 inhibition in ovarian cancer were elucidated by using *VEGFR2*^*Y949F/Y949F*^ and MISIIR-TAg spontaneous epithelial ovarian carcinoma mouse models.

## Materials and methods

### Antibodies

Antibodies against ANGPTL4 (ab196746; also used for immunofluorescence at a 1/100 dilution), VE-cadherin (ab33168), and GAPDH (ab8245) for western blotting were obtained from Abcam (Cambridge, UK) and used at a 1/1000 dilution; antibodies against slug (9585), snail (3879), VEGFR2 (9698; also used for immunoprecipitation), phospho-Src Y416 (2101), Src (2109) and phospho-VEGFR2 Y949 (2471; human Y951, corresponding to mouse Y949) for western blotting were purchased from Cell Signaling Technology (Danvers, MA, USA) and used at a 1/1000 dilution. Antibodies against CD31 (ab28364) and VE-cadherin (ab33168) for immunofluorescence were purchased from Abcam and used at a 1/100 dilution. Antibodies against SV40 large T antigen (15,729) for immunofluorescence were acquired from Cell Signaling Technology and used at a 1/100 dilution.

### Ovarian cancer cell lines and cell culture

Human ovarian clear cell carcinoma ES2 and human ovarian adenocarcinoma COC1, SKOV3, OVCAR3, and CAOV3 cells were obtained from the American Type Culture Collection (ATCC; Manassas, VA, USA). Human ovarian carcinoma A2780 cells were purchased from Sigma-Aldrich, and NCI/ADR-RES cells were purchased from the National Cancer Institute Division of Cancer Treatment and Diagnosis (Bethesda, MD, USA). ES2, COC1, SKOV3, OVCAR3, and CAOV3 cells were cultured in Dulbecco’s modified Eagle’s medium (DMEM) supplemented with 10% filtered fetal bovine serum (FBS); A2780 and NCI/ADR-RES cells were maintained in Roswell Park Institute Medium-1640 (RPMI-1640) supplemented with 10% FBS (all from Gibco; Grand Island, NY, USA). All cell lines were cultured at 37 °C in a humidified environment containing 5% CO_2_/95% air.

### RNA interference (RNAi)

Human ANGPTL4 small interfering RNA (siRNA) and nontargeting scrambled control siRNA were constructed using pGCSIL-GFP (Addgene; Watertown, MA, USA). Lentiviruses containing GFP and siRNA sequences were produced and purified by GeneChem (Shanghai, China). For ANGPTL4 interference in ovarian cancer cell lines, cells were transduced with modified lentiviral expression vectors following the manufacturer’s protocol. Cells were harvested 72 h post transduction for in vivo injection or cytological analysis. siRNA sequences are shown in Additional file 1: Table S1.

### Mice and xenograft model

Female athymic BALB/c nude mice (5–6 weeks old) were obtained from The Jackson Laboratory (Bar Harbor, ME, USA) and socially housed in a pathogen-free facility. Normal or lentiviral vector-transduced A2780 and CAOV3 ovarian cancer cell lines (1 × 10^7^ cells in 200 μL of PBS) were subcutaneously injected into the left axilla of nude mice. Tumor-bearing mice were monitored by the body condition score (BCS) index and clinical evaluation and were euthanized by using an overdose of pentobarbital sodium (100 mg/kg, i.v.) if they showed either a > 10% increase in body weight compared with the preinjection body weight or if their activity level declined due to tumor burden. Mice were sacrificed with 100 mg/kg pentobarbital sodium 30 days post injection to harvest the tumors. Tumor volumes were estimated with the formula length × width^2^ × 0.5. Animal procedures were approved by the Animal Care and Use Committee of Naval Military Medical University and were conducted in accordance with its ethical standards.

### *VEGFR2*^*Y949F/Y949F*^ and MISIIR-TAg spontaneous epithelial ovarian carcinoma mouse models.

A mouse model with phenylalanine (F) knock-in to replace the tyrosine (Y) at position 949 (Y951 in humans) of VEGFR2 (*VEGFR2*^*Y949F/Y949F*^) on a mixed 129S6/C57BL/6 background was established using VelociGene technology (Regeneron Pharmaceuticals; Tarrytown, NY, USA) [22]. *VEGFR2*^*Y949F/Y949F*^ mice were then backcrossed onto the C57BL/6 background for 12 generations, and homozygous mice were obtained by crossing *VEGFR2*^*Y949F/Y949F*^ heterozygotes. Wild-type female C57BL/6 mice were utilized as WT mice.

MISIIR-TAg transgenic mice (TAg mice) were generated as previously described by Denise Connolly [23]; these mice express SV40 T antigen (SV40-TAg) under the Mullerian inhibitory substance type II receptor (MISIIR) promoter and are susceptible to spontaneous ovarian carcinoma. *VEGFR2*^Y949F/Y949F^ mice were crossed with TAg mice on a C57BL/6 background to generate TAg-WT and TAg-*VEGFR2*^Y949F/Y949F^ homozygous mice with induced spontaneous bilateral epithelial ovarian carcinoma and/or continuous inactivation of VEGFR2 phosphorylation at Y949. For all experimental groups, other than when specifically noted, WT and Y949F indicate TAg-WT and TAg-*VEGFR2*^Y949F/Y949F^ mice, respectively.

After sex selection at birth, tail biopsies from female pups were collected for genotype confirmation. On postnatal day 28, the mice underwent laparotomy, and lentiviral siRNA targeting ANGPTL4 (3 × 10^9^ PFU) was delivered by direct intraovarian injection into the bilateral ovaries using a G30 Hamilton syringe mounted on a screw-actuated micrometer device (Harvard apparatus; Holliston, MA, USA). On postnatal day 56, mice were sacrificed for further analysis, and ANGPTL4 knockdown was confirmed, as shown in Additional file 1: Fig. S1a, b. Tumor volumes were calculated using hematoxylin and eosin-stained sections obtained from throughout the ovaries. The two largest sections of each tumor were used for quantification of vessel density and proliferative activity.

### Western blotting

Tissues and cell lysates were prepared with RIPA buffer (Beyotime; Shanghai, China) supplemented with 1 mM phenylmethylsulfonyl fluoride, and protein concentrations were measured by using a BCA Kit (Beyotime). After separating proteins in the samples by sodium dodecyl sulfate–polyacrylamide gel electrophoresis (Beyotime), the proteins were transferred onto polyvinylidene difluoride membranes (Millipore; Burlington, MA, USA). Membranes were blocked with 5% skim milk for 2 h at room temperature and incubated with primary antibodies overnight at 4 °C. After incubation with the appropriate horseradish peroxidase-linked secondary antibodies, immunoreactions on the membranes were detected with an enhanced chemiluminescence detection kit (Beyotime). Digitized signals were analyzed using ImageJ 1.53 software (National Institutes of Health; Bethesda, MD, USA). GAPDH was used as the endogenous control.

### Immunoprecipitation

For immunoprecipitation, tissue lysates were obtained with RIPA buffer and precleared by incubation with 25 mL of Protein G Sepharose 4 Fast Flow beads (Ge Healthcare, 17–0618-01; Chicago, IL, USA) per sample for 2 h at 4 °C. Precleared lysates were incubated with the appropriate VEGFR2 antibody (approximately 4 μL of the antibody was used for 400 μg of total protein) and 25 ml of Protein A/G beads (Santa Cruz, sc-2003; Dallas, TX, USA) overnight at 4 °C. The Sepharose beads were washed 5 times with 1 ml RIPA buffer per sample and collected by centrifugation at 3000 × g for 1 min at 4 °C. Immunocomplexes were processed for immunoblotting as described above. Normal mouse IgG (Santa Cruz, sc-2025) was used as the negative control.

### Quantitative reverse transcription PCR (qRT-PCR)

Total RNA was isolated from tumor cell lines or tumor tissues using an RNeasy Mini Kit (Qiagen, 74,104; Germantown, MD, USA) with DNase I treatment (Qiagen, 79,254). cDNA was generated by reverse transcription with SuperScript II Reverse Transcriptase (Thermo Fisher, 18,064; Waltham, MA, USA). qPCR was performed in triplicate utilizing Power SYBR Green PCR Mix (Applied Biosystems; Foster City, CA, USA), and mRNA levels were normalized to those of GAPDH by using the comparative Ct (2^−ΔΔCt^) method [24]. Primer sequences are shown in Additional file 1: Table S1.

### Immunofluorescence staining

Tumor tissues were isolated and frozen in O.C.T. (Sakura Finetek; Yokohama, Kanagawa, Japan), and cryosections (8 μm) were sliced. Cryosections or ovarian cancer cells were prefixed in 4% paraformaldehyde for 20 min at room temperature. After permeabilization with 0.5% Triton X-100 (Sigma-Aldrich; St. Louis, MO, USA), 5% donkey serum was used for blocking for 1 h at room temperature. Immunostaining was performed using primary antibodies overnight at 4 °C, and samples were subsequently treated with Alexa Fluor-conjugated secondary antibodies (Jackson ImmunoResearch Laboratories; West Grove, PA, USA) for 1 h at room temperature. Nuclei were counterstained with Hoechst 33,258 (Sigma-Aldrich, 14,530). Images were acquired using a Zeiss LSM 700 confocal microscopy system (Carl Zeiss Jena, Oberkochen, Germany) and quantified with ImageJ software. Skeletonization of CD31 staining was performed and vessel length was measured as previously described [25].

### Transwell assays

Transwell invasion assays and migration assays were conducted in a 24-well Transwell chamber system with 8 µm pore-size membranes (Corning; Corning, MA, USA). A2780 or CAOV3 ovarian cancer cells (1 × 10^5^) were seeded into the upper compartments with or without Matrigel coating on the membrane (Corning) in 200 μL of serum-free medium. A total of 700 μL of culture medium containing 10% FBS was added into the lower compartments as an attractant. After culture for 12 h at 37 °C, cells were fixed with 4% paraformaldehyde, stained with crystal violet (Sigma-Aldrich, C0775) and counted under a light microscope. The number of cells counted in six random fields was averaged.

### Cell viability and proliferation assays

Ovarian cancer cell viability was analyzed by crystal violet staining. Briefly, cells were prefixed with 4% paraformaldehyde for 20 min at room temperature. Crystal violet (0.5%) was then added for 30 min at room temperature, and the cells were photographed. Cell proliferation was evaluated by a CCK-8 assay (Beyotime, Jiangsu, China) at time points up to 96 h after cell plating. Briefly, 5 × 10^3^ cells were plated into 96-well plates (Corning), and 10 μL of CCK-8 solution was added to each well and incubated for 1 h at 37 °C. The absorbance at 450 nm was detected using a microplate reader (Synergy 4, BioTek; Winooski, VT, USA).

For the 5-ethynyl-2′-deoxyuridine (EdU) incorporation assay, cells were seeded at 30% confluence in 6-well plates after 48 h of transduction and were continuously cultured for 24 h. After incubation with 50 μM EdU (Sigma-Aldrich, 900,584) for 2 h, cells were fixed with 4% paraformaldehyde and analyzed by using an iClick EdU Andy Fluor 488 Flow Cytometry Assay Kit (ABP Biosciences, Beltsville, MD, USA). For the in vivo EdU incorporation assay, tumor-bearing, TAg-WT, and TAg-Y949F mice were intravenously injected via the tail vein with 100 μg of EdU 24 h before sacrifice. EdU incorporation into newly synthesized DNA was also measured in vivo. Hoechst 33,258 was used to stain nucleic acids. The number of EdU-positive cells was counted.

### Statistical analysis

Parametric data were analyzed by Student’s t-test and one-way ANOVA followed by Tukey’s honest significant difference (HSD) test for comparisons between 2 treatment groups or among more than 2 treatment groups, respectively. The Sidak test was performed when individual time points were compared to each other. Nonparametric data were compared with the Mann–Whitney U-test/Wilcoxon signed-rank test (for paired comparisons) or with the Kruskal–Wallis test followed by Dunn’s multiple comparison test for comparisons between 2 groups or among more than 2 groups, respectively. GraphPad Prism 8.0 (GraphPad Software, Diego, CA, USA) was used for statistical analyses. *p* values < 0.05 were considered statistically significant (*p < 0.05, **p < 0.01, ***p < 0.001).

## Results

### ANGPTL4 is upregulated in specific ovarian cancer cell lines

**Fig. 1:**
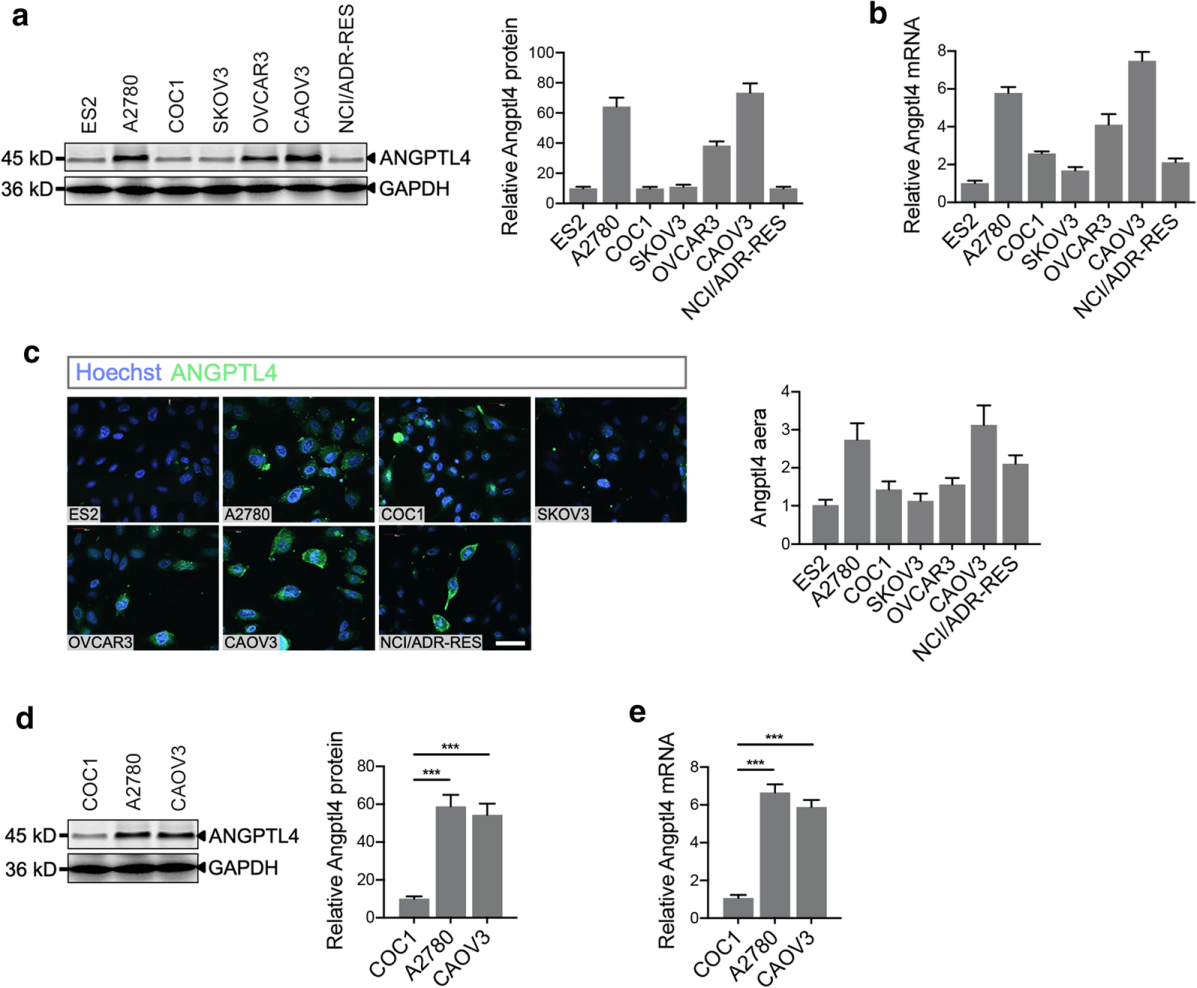
ANGPTL4 expression is highest in A2780 and CAOV3 ovarian cancer cells and tumors. **a** Western blot and statistical graph of angiopoietin-like 4 (ANGPTL4) expression in human ovarian clear cell carcinoma ES2 cells, human ovarian carcinoma A2780 and NCI/ADR-RES cells, and human ovarian adenocarcinoma COC1, SKOV3, OVCAR3, and CAOV3 cells; n = 3 independent experiments per group. **b** qRT-PCR analysis was performed to determine the expression pattern of ANGPTL4 mRNA in human ovarian cancer cells; n = 3 independent experiments per group. **c** Immunofluorescence analysis and statistical graph of ANGPTL4 expression in ovarian cancer cells; n = 3 per group. Scale bar: 50 μm. **d** Western blot and statistical graph of ANGPTL4 expression in the A2780 and CAOV3 xenograft models; n = 4 independent experiments per group. **e** qRT-PCR analysis of ANGPTL4 mRNA expression was also carried out in A2780 and CAOV3 tumors; n = 4 independent experiments per group. ***p < 0.001 for the indicated comparisons. The data are presented as the mean ± SEM values

ANGPTL4 expression was measured in human ovarian clear cell carcinoma ES2, human ovarian carcinoma A2780 and NCI/ADR-RES, and human ovarian adenocarcinoma COC1, SKOV3, OVCAR3, and CAOV3 cells. As shown in Fig. 1a, we found that A2780, OVCAR3, and CAOV3 cells expressed the highest levels of the ANGPTL4 protein, whereas ES2, COC1, SKOV3, and NCI/ADR-RES cells expressed low to negligible levels. qRT-PCR analysis also revealed high ANGPTL4 mRNA levels in A2780, OVCAR3, and CAOV3 cells (Fig. 1b). Further evidence was provided by an immunofluorescence assay, which suggested high ANGPTL4 expression in A2780 and CAOV3 cells (Fig. 1c). Subsequently, COC1, A2780, and CAOV3 cells (1 × 10^7^ cells) were subcutaneously injected into the left axilla of female BALB/c nude mice, and the tumors were harvested for ANGPTL4 expression analysis (Fig. 1d, e). High levels of ANGPTL4 protein and mRNA were also observed in A2780 and CAOV3 xenograft mice compared to COC1 mice.

### ANGPTL4 knockdown in A2780 and CAOV3 cells by human ANGPTL4 siRNA targeting ANGPTL4

**Fig. 2:**
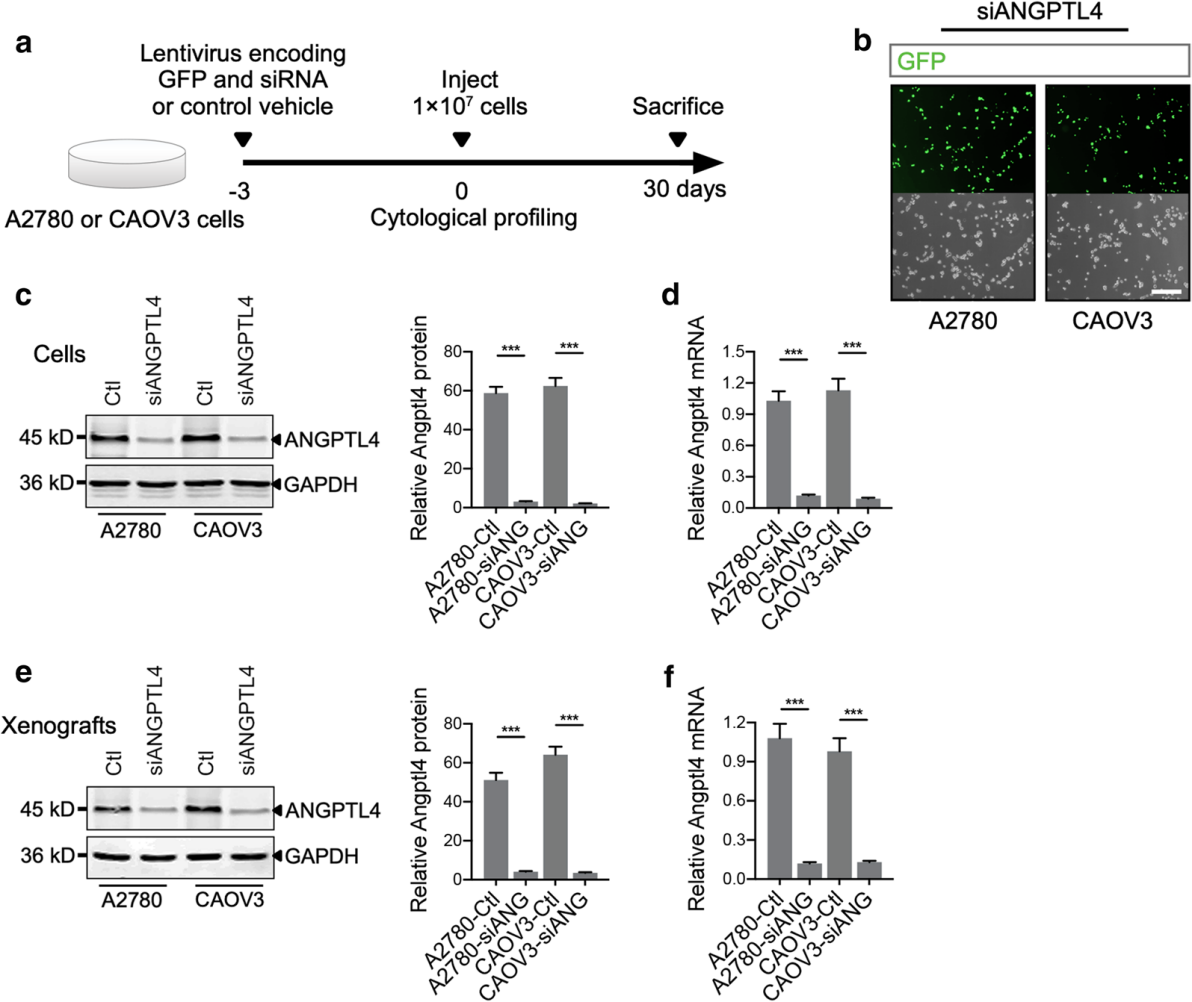
ANGPTL4 downregulation in A2780 and CAOV3 ovarian cancer cells through RNA interference (RNAi) technology. **a** Experimental strategy. A2780 or CAOV3 cells were transduced with lentiviral vectors encoding GFP and siRNA targeting ANGPTL4 (siANG) or scrambled control siRNA (Ctl) for 3 days and were then subjected to cytological profiling. Then, 1 × 10^7^ cells were injected into the left axilla of 5- to 6-week-old female BALB/c nude mice. Thirty days after injection, mice were sacrificed to harvest the tumors for further analysis. **b** Immunofluorescence of green fluorescence protein (GFP) and bright field images of A2780 and CAOV3 cells confirmed the transduction efficiency of the lentiviral vectors. Scale bars: 75 μm. **c** Western blot and statistical graph of ANGPTL4 expression in A2780 and CAOV3 cells transduced with siANG or Ctl; n = 3 independent experiments per group. **d** qRT-PCR to determine the ANGPTL4 mRNA level in A2780 and CAOV3 cells transduced with lentiviral vectors; n = 3 independent experiments per group. **e** Western blot and statistical graph of ANGPTL4 expression in transduced A2780 and CAOV3 tumor xenograft models; n = 5 independent experiments per group. **f** qRT-PCR to verify ANGPTL4 mRNA expression in transduced A2780 and CAOV3 tumors; n = 5 independent experiments per group. ***p < 0.001 for the indicated comparisons. The data are presented as the mean ± SEM values. siANG, small interfering RNA targeting ANGPTL4

Next, human ANGPTL4 siRNA (siANGPTL4) and nontargeting scrambled control siRNA (Ctl) were inserted into the pGCSIL-GFP lentiviral expression vector and transduced into A2780 or CAOV3 cells 3 days before in vivo injection (Fig. 2a). GFP fluorescence combined with cellular bright field microscopy showed that the transduction efficiency was ~ 57% in A2780 cells and ~ 61% in CAOV3 cells (Fig. 2b). Furthermore, ANGPTL4 expression in vitro was analyzed through immunoblotting (Fig. 2c) and qRT-PCR (Fig. 2d). Significant inhibition of ANGPTL4 was observed in siANGPTL4-transduced A2780 and CAOV3 cells compared with the corresponding Ctl cells. Decreased ANGPTL4 expression was also confirmed in A2780 and CAOV3 xenograft tumors 30 days after in vivo injection, indicating long-lasting lentivirus-based RNA suppression (Fig. 2e, f).

### Cell proliferation, migration, and invasion were not affected in ovarian cancer cells with ANGPTL4 inhibition

**Fig. 3:**
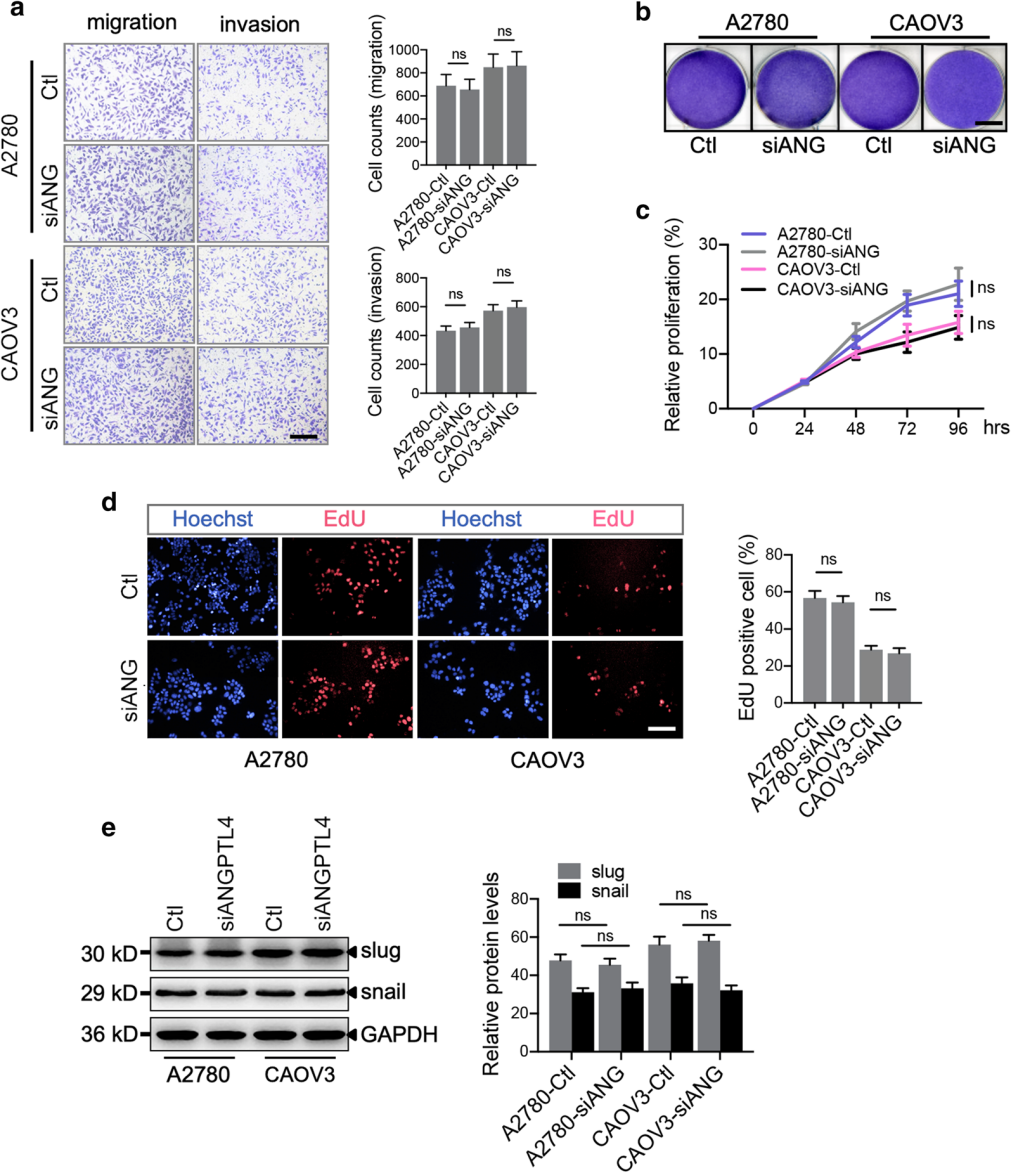
ANGPTL4 knockdown does not alter ovarian cancer cell proliferation, migration or invasion in vitro. **a** Transwell assay and statistical graph of the migration and invasion of A2780 and CAOV3 cells with or without ANGPTL4 inhibition; n = 4 independent experiments per group. Scale bar: 100 μm. **b** Crystal violet staining of transduced A2780 and CAOV3 cells. Scale bar: 12 mm. **c** A2780 and CAOV3 cell proliferation was investigated by a CCK-8 assay; n = 3 independent experiments per group. **d** Fluorescence staining and statistical graph of 5-ethynyl-2′-deoxyuridine (EdU) incorporation in transduced ovarian cancer cells; n = 3 independent experiments per group. Scale bar: 80 μm. **e** Western blot of the epithelial-mesenchymal transition (EMT)-related markers slug and snail in transduced ovarian cancer cells; n = 3 independent experiments per group. ns indicates no significance. The data are presented as the mean ± SEM values

ANGPTL4 not only regulates vascular biology but also is related to other cellular metabolic processes, such as lipid metabolism [26]; thus, whether ANGPTL4 inhibition affects biological processes in ovarian cancer cell lines (A2780 and CAOV3) was investigated. Transwell assays demonstrated that silencing ANGPTL4 did not affect the migration and invasion abilities of ovarian cancer cells (Fig. 3a). Additionally, crystal violet staining and CCK-8 assays revealed that ANGPTL4 knockdown had no effect on cell proliferation in vitro up to 96 h after transduction, consistent with the EdU incorporation assay results (Fig. 3b–d). Western blotting was conducted to evaluate epithelial-mesenchymal transition (EMT) marker expression. There was no difference in slug and snail expression between the siANGPTL4 and scrambled Ctl groups in either A2780 or CAOV3 cells (Fig. 3e). Our results indicated that ANGPTL4 silencing did not inhibit ovarian cancer cell metastasis in vitro.

### Silencing ANGPTL4 reduces the tumor burden in mice bearing A2780 and CAOV3 xenografts

**Fig. 4:**
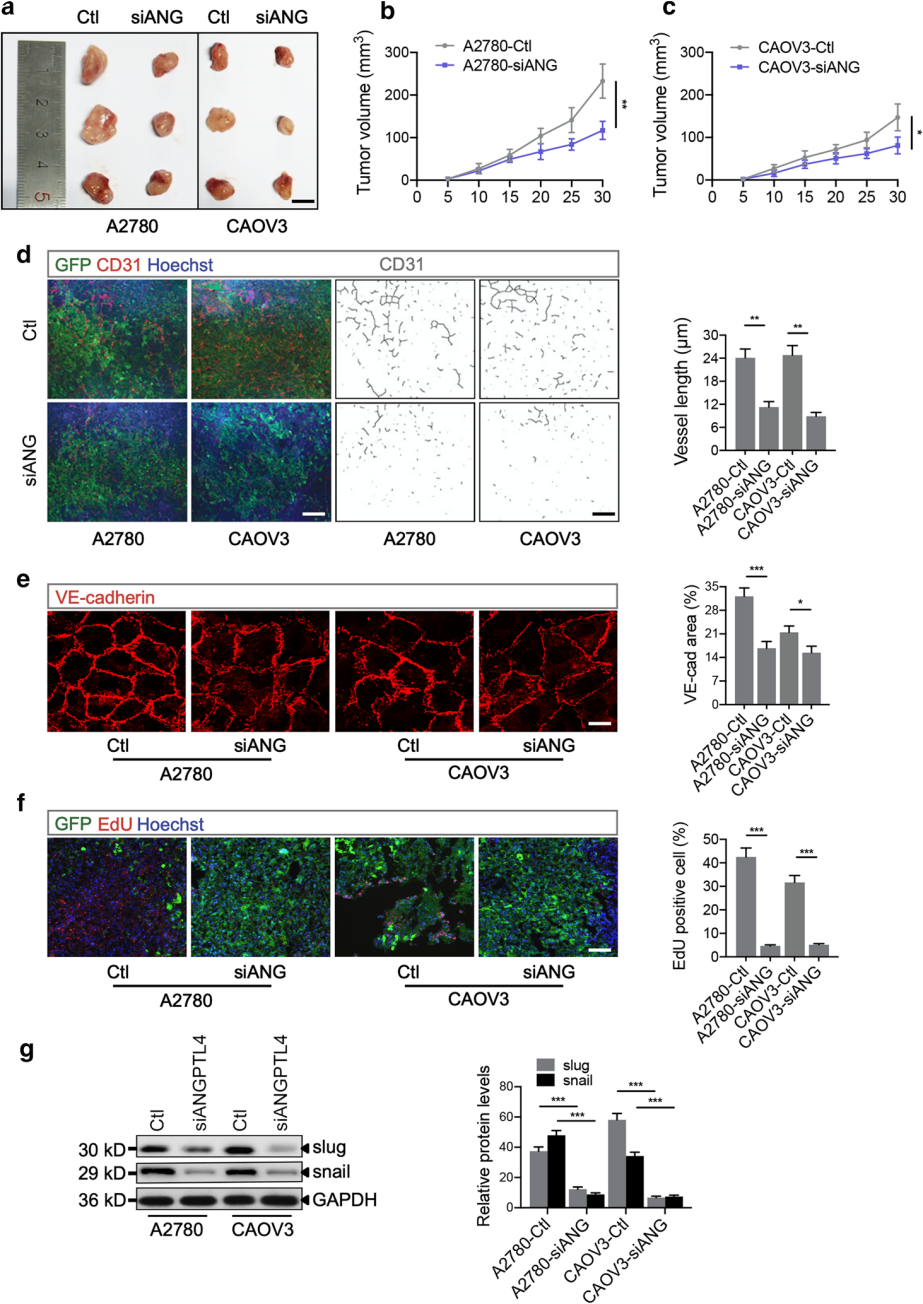
Decreased ANGPTL4 expression inhibits human ovarian cancer xenograft growth in nude mice. **a**–**c** Xenograft tumors of nude mice 30 days after injection of transduced A2780 or CAOV3 cells; n = 3 independent experiments per group. Scale bar: 7 mm. Total A2780 and CAOV3 tumor volumes were determined every 5 days. **d** GFP and CD31 immunofluorescence staining of A2780 and CAOV3 tumors (left) and skeletonization of CD31 staining to quantify total vessel length in the tumor vasculature in each field (right). Quantification of vessel length according to skeletonization of CD31 staining; n = 3 independent experiments per group. Scale bar: 100 μm. **e** Immunofluorescence staining and statistical graph of VE-cadherin expression in A2780 and CAOV3 tumors; n = 3 independent experiments per group. Scale bar: 10 μm. **f** Fluorescence staining and statistical graph of EdU incorporation in A2780 and CAOV3 tumors; n = 3 independent experiments per group. Scale bar: 100 μm. **g** Western blot and statistical graph of slug and snail expression in A2780 and CAOV3 tumors; n = 4 independent experiments per group. *p < 0.05, **p < 0.01, ***p < 0.001 for the indicated comparisons. The data are presented as the mean ± SEM values

Transduced A2780 and CAOV3 ovarian cancer cells were then xenografted into nude mice to identify the roles of ANGPTL4 in ovarian tumorigenesis in vivo. Importantly, the total tumor volume in mice bearing siANGPTL4 A2780 or CAOV3 xenografts were significantly reduced compared to those in the corresponding Ctl mice (Fig. 4a–c). The tumor vasculature and total vessel length in A2780 and CAOV3 tumors were evaluated by CD31 staining and CD31-based skeletonization (Fig. 4d). The results suggested that siANGPTL4 decreased the vessel length in A2780 and CAOV3 tumors compared with the corresponding Ctl tumors. Importantly, disorganization of VE-cadherin accompanied by decreased expression was observed in A2780 and CAOV3 tumors with ANGPTL4 knockdown in comparison with the corresponding Ctl tumors (Fig. 4e). Moreover, fewer EdU-positive cells (Fig. 4f) and lower EMT marker (slug and snail) expression (Fig. 4g) were observed in A2780 and CAOV3 tumors with ANGPTL4 knockdown, indicating inhibition of ovarian cancer cell proliferation and invasion. These results showed that ANGPTL4 knockdown reduced the tumor volume, degenerated the tumor vasculature, and inhibited tumor metastasis, which revealed the great promise of ANGPTL4 inhibitors for ovarian cancer therapy.

### Dissociation of the VEGFR2/VE-cadherin/Src complex and phosphorylation of VEGFR2 at Y949 are responsible for the tumor-suppressive effects of ANGPLT4 inhibition

**Fig. 5:**
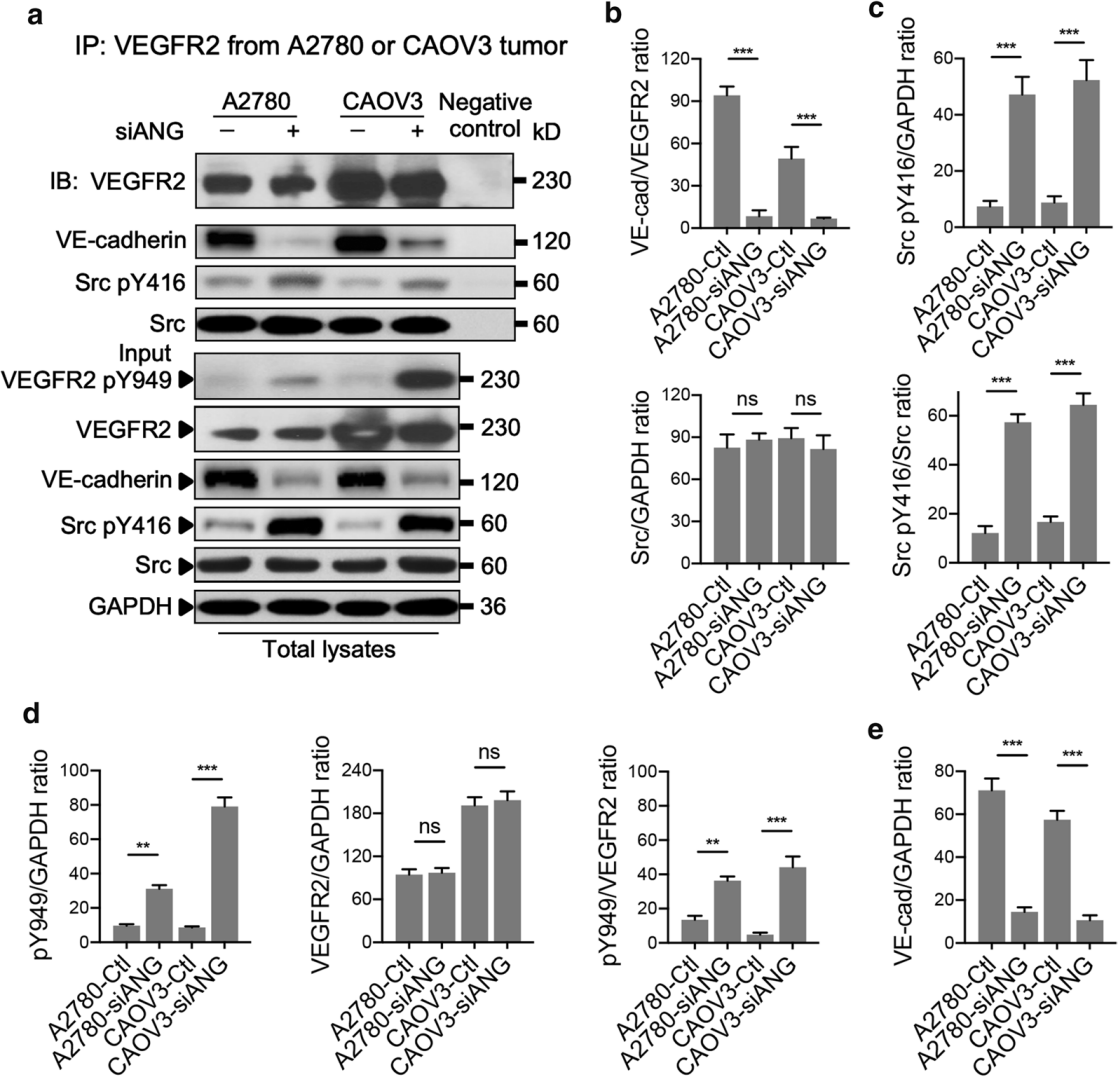
Analysis of the VEGFR2/VE-cadherin/Src complex in A2780 and CAOV3 tumors treated with or without siRNA targeting ANGPTL4. **a** Immunoprecipitation (IP) of vascular endothelial growth factor receptor 2 (VEGFR2) and immunoblotting (IB) of VE-cadherin, Src kinase phosphorylated at tyrosine 416 (Src pY416), Src, and VEGFR2. Total lysates were immunoblotted for VEGFR2 phosphorylated at tyrosine 949 (VEGFR2 pY949), VEGFR2, VE-cadherin, Src pY416, Src, and GAPDH; n = 3 independent experiments per group. **b** VE-cadherin/VEGFR2 ratio in immunoprecipitates from A2780 and CAOV3 tumors. **c** The Src pY416 level normalized to that of GAPDH or Src and the Src/GAPDH ratio were determined in immunoblots of A2780 and CAOV3 total lysates. **d** The VEGFR2 pY949 level normalized to that of GAPDH or VEGFR2 and the VEGFR2/GAPDH ratio were analyzed in total lysates by immunoblotting. **e** VE-cadherin/GAPDH ratio in immunoblots of A2780 and CAOV3 total lysates. **p < 0.01, ***p < 0.001 for the indicated comparisons. ns indicates no significance. The data are presented as the mean ± SEM values

To further investigate the mechanism leading to disorganization of VE-cadherin and reduced angiogenesis in A2780 and CAOV3 tumors with ANGPTL4 knockdown, Src kinase and VE-cadherin signaling downstream of VEGFR2 was analyzed. Tumor lysates were immunoprecipitated for VEGFR2 and were then immunoblotted for VEGFR2, VE-cadherin, phospho-Src at tyrosine 416 (Src pY416), and Src (Fig. 5a). Additionally, immunoblotting for VEGFR2 phosphorylated at tyrosine 949 (VEGFR2 pY949), VEGFR2, VE-cadherin, Src pY416, Src, and GAPDH in total lysates was used as an input control. Transient destabilization of VE-cadherin and dissociation of the VEGFR2/Src complex was observed in A2780 and CAOV3 tumors with ANGPTL4 knockdown, accompanied by increased Src pY416 levels (Fig. 5b, c). In addition, phosphorylation of VEGFR2 at Y949 was upregulated in ANGPLT4 knockdown tumors in comparison with the corresponding Ctl tumors (Fig. 5d). Furthermore, total VE-cadherin levels in ANGPTL4 knockdown tumor lysates were decreased compared to those in the corresponding Ctl tumors (Fig. 5e). No difference was observed in the expression of Src kinase and VEGFR2 between tumors generated from siANGPTL4- and scrambled Ctl -transduced cells (Fig. 5c, d). These results suggested that increases in the phosphorylation of VEGFR2 at Y949 and Src kinase at Y416 combined with a decrease in the expression of VE-cadherin resulted in dissociation of the VEGFR2/VE-cadherin/Src complex. Both events are responsible for VE-cadherin reduction and decreased tumor vascularization in mice bearing ANGPLT4-silenced A2780 and CAOV3 xenografts.

### Inactivation of VEGFR2 phosphorylation at Y949 eliminates the tumor-suppressive effects of siANGPTL4 in *VEGFR2*^*Y949F/Y949F*^ mice

**Fig. 6:**
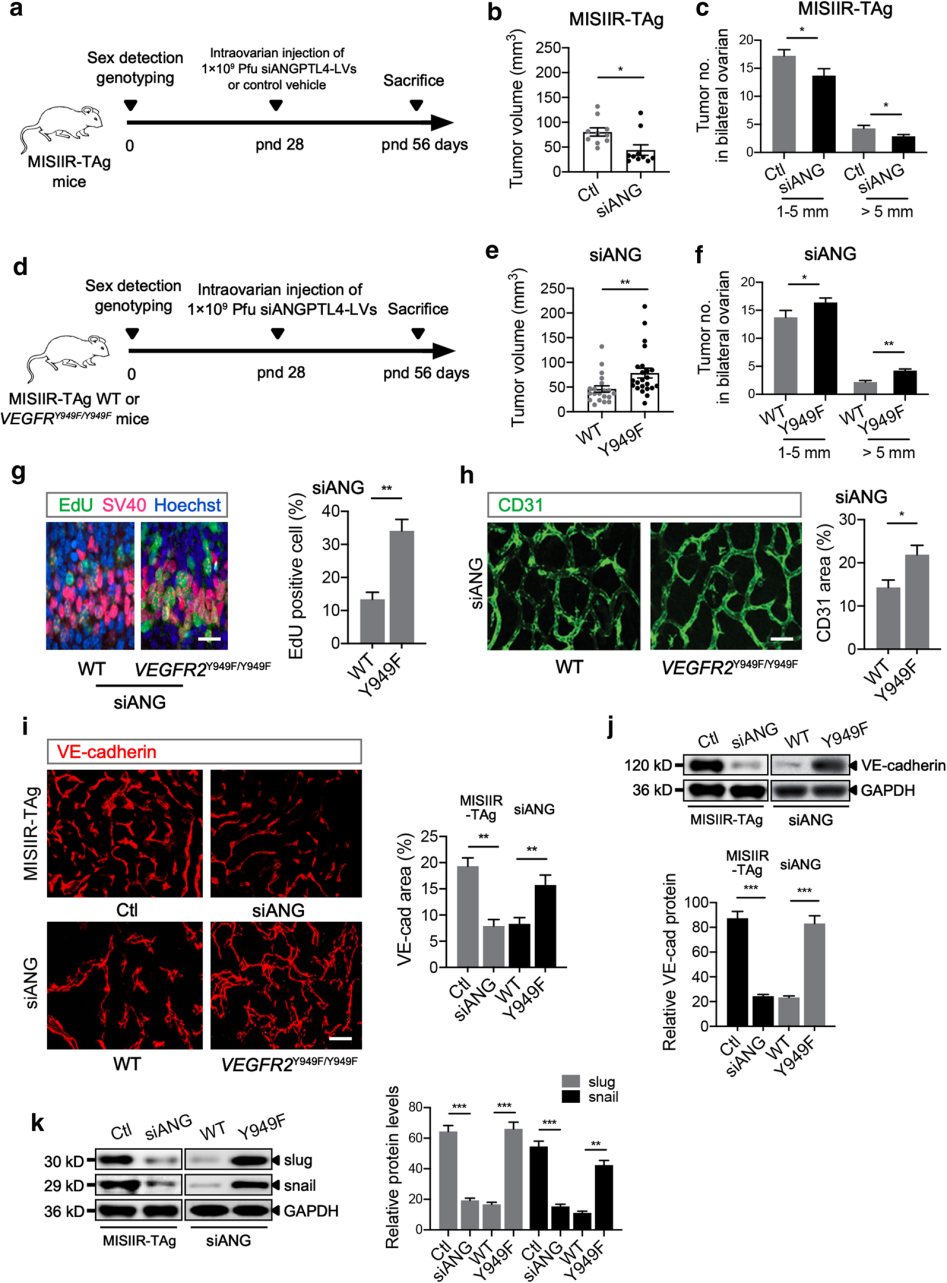
ANGPTL4 knockdown loses its tumor-suppressive effect in *VEGFR2 *^*Y949F/Y949F*^ mice. **a** Experimental strategy. MISIIR-TAg mice (TAg mice) were generated, the sex of the pups was determined, and tail tips were acquired from female mice for genotyping. On postnatal day (pnd) 28, with control lentiviral vectors (LVs) or LVs encoding siRNA targeting ANGPTL4 were injected into the ovaries of mice. On pnd 56, mice were sacrificed for further analysis. **b** Ovarian tumor volumes in control (Ctl)-injected and siANGPTL4 (siANG)-injected mice on pnd 56; n = 10 mice per group. **c** Distribution of tumor diameters (1–5 mm and > 5 mm) on pnd 56; n = 10 mice per group. **d** Experimental strategy. *VEGFR2*^*Y949F/Y949F*^ mice were crossed with MISIIR-TAg mice on a C57BL/6 background to obtain TAg-WT (WT) and TAg-*VEGFR2*^*Y949F/Y949F*^ (*VEGFR2*^*Y949F/Y949F*^ or Y949F) homozygous mice with induced spontaneous epithelial ovarian carcinoma and/or continuous inactivation of VEGFR2 at tyrosine 949. On pnd 28, LVs encoding siRNA targeting ANGPTL4 were injected into the ovaries of mice. On pnd 56, mice were sacrificed for further analysis. **e** WT and *VEGFR2*^*Y949F/Y949F*^ ovarian tumor volumes on pnd 56; n = 20–23 mice per genotype. **f** Distribution of tumor diameters (1–5 mm and > 5 mm) on pnd 56; n = 20–23 mice per genotype. **g** EdU-positive (green) cell/simian virus 40 large T antigen (SV40, red)-positive tumor cell ratio in ovarian tissue; n = 7 tumors per genotype. Scale bar: 30 μm. **h** Immunofluorescence staining and statistical graph of CD31 expression in WT and *VEGFR2*^*Y949F/Y949F*^ ovarian tumors; n = 6 tumors per genotype. Scale bar: 50 μm. **i** Immunofluorescence staining and statistical graph of VE-cadherin expression in Ctl and siANG tumors or WT and *VEGFR2*^*Y949F/Y949F*^ tumors; n = 4–6 tumors per group. Scale bar: 50 μm. **j** Western blot of VE-cadherin and GAPDH in Ctl and siANG tumors or WT and *VEGFR2*^*Y949F/Y949F*^ tumors; n = 4 tumors per group. **k** Western blot of slug, snail, and GAPDH in Ctl and siANG tumors or WT and *VEGFR2*^*Y949F/Y949F*^ tumors; n = 4 tumors per group. *p < 0.05, **p < 0.01, ***p < 0.001 for the indicated comparisons. The data are presented as the mean ± SEM values

To further determine the effects of ANGPTL4 on ovarian cancer, an orthotopic ovarian cancer model was established, and control or siANGPTL4 lentiviral vectors were injected into the ovaries of MISIIR-TAg mice (Fig. 6a). The total tumor volumes were significantly reduced in siANGPTL4-treated mice (Fig. 6b). Similarly, siANGPTL4-injected mice showed fewer small (1–5 mm) and large (> 5 mm) ovarian tumors than control-injected mice (Fig. 6c). As VEGFR2 Y949 plays an important role in tumor vascularization and growth [19], this tyrosine (Y) in VEGFR2 was mutated to phenylalanine (F) and spontaneous ovarian carcinoma was induced in siANGPTL4-injected mice to further determine the connection between ANGPTL4 and VEGFR2 pY949 in tumors. TAg-WT and TAg-*VEGFR2*^*Y949F/Y949F*^ homozygous mice on a C57BL/6 background were generated by crossing MISIIR-TAg and *VEGFR2*^*Y949F/Y949F*^ mice and were used in this study after sex selection, sequence verification and intraovarian injection of control (Additional file 1: Fig. S2a) or siANGPTL4 lentiviral vectors (Fig. 6d). The total tumor volumes and tumor numbers were significantly increased in siANGPTL4-injected TAg-*VEGFR2*^*Y949F/Y949F*^ mice compared to TAg-WT mice (Fig. 6e, f), and no difference was observed between vehicle control-injected TAg-WT and TAg-*VEGFR2*^*Y949F/Y949F*^ mice (Additional file 1: Fig. S2b, c). Examination of ovaries demonstrated that the EdU-positive cell/SV40-positive cell ratio in TAg-*VEGFR2*^*Y949F/Y949F*^ mice was significantly higher than that in TAg-WT mice on day 56 (Fig. 6g). Furthermore, the connection between VEGFR2 pY949 and tumor angiogenesis was investigated. As shown in Fig. 6h, the tumor vascular density in TAg-*VEGFR2*^*Y949F/Y949F*^ mice was significantly elevated compared with that in TAg-WT mice. Importantly, VE-cadherin was preferentially localized at endothelial junctions in the tumor vasculature in TAg-*VEGFR2*^*Y949F/Y949F*^ mice, and the VE-cadherin expression levels in tissue sections and total lysates were significantly higher than those in TAg-WT mice. Additionally, a decrease in VE-cadherin expression was observed in siANGPTL4-injected mice compared with control-injected mice (Fig. 6i, j). The expression of EMT markers in siANGPTL4-injected mice was downregulated compared with that in control-injected mice, and inactivation of VEGFR2 phosphorylation at Y949 significantly increased the slug and snail levels (Fig. 6k). We did not observe changes in the expression of VE-cadherin or EMT-related proteins between TAg-WT and TAg-*VEGFR2*^*Y949F/Y949F*^ mice injected with vehicle control (Additional file 1: Fig. S2d-f).

## Discussion

Here, we show that specific blockade of ANGPTL4-induced tumor angiogenesis results in inhibition of ovarian cancer cell proliferation and tumor growth in vivo. Our results show that the expression of ANGPTL4 is different in several ovarian cancer cell lines and that A2780 and CAOV3 cells have the highest expression of ANGPTL4. Silencing ANGPTL4 reduces A2780 and CAOV3 tumor growth, angiogenesis, and metastasis in vivo but not in vitro, indicating that ANGPTL4 is essential for tumor proliferation and vascular angiogenesis in ovarian cancer. However, several studies have claimed that ANGPTL4 inhibits vascular angiogenesis and prevents tumor metastasis in melanoma B16F0, lung cancer 3LL [10] and colon cancer CMT93 [9] xenograft mouse models; however, contrasting results were observed in breast cancer [11], renal cell carcinoma [14], and ovarian cancer [16, 17] xenograft models, indicating that ANGPTL4 may act as a tissue-specific and multifunctional vascular modulator. Furthermore, previous studies have shown that two proteins, PDZ-binding motif (TAZ) and testisin, inhibit ovarian cancer metastasis and that ferroptosis and chemoresistance are also related to ANGPTL4 inhibition [16, 27].

Perdiguero and coworkers reported the critical role of ANGPTL4 in vascular integrity through the VEGFR2-VE-cadherin interaction and VEGFR2-Src kinase signaling [28]. Our work extends these findings by implicating the VEGFR2 pY949/VE-cadherin/Src pY416 complex in the modulation of vascular integrity. In the present study, the immunoprecipitation and immunoblot analysis results suggest that deletion of ANGPTL4 leads to dissociation of the VEGFR2/VE-cadherin/Src complex and degrades VE-cadherin via phosphorylation of VEGFR2 Y949 and Src Y416. Inactivation of VEGFR2 Y949 phosphorylation in TAg-*VEGFR2*^*Y949F/Y949F*^ mice abolishes all effects of siANGPTL4 on the inhibition of tumor proliferation and metastasis and the suppression of vascular angiogenesis, suggesting that VEGFR2 pY949 is responsible for the tumor-suppressive effects of siANGPTL4 in ovarian cancer.

It should be noted, however, that as VE-cadherin is a component of intercellular adherens junctions in endothelial cells and plays an important role in regulating vascular permeability [29], downregulation of VE-cadherin in endothelial junctions by siANGPTL4 may increase tumor vascular permeability and subsequently increase the risk of tumor metastasis and spread [30]. In this study, primary tumor growth and proliferation were significantly inhibited with siANGPTL4 treatment, and the levels of EMT markers were reduced, suggesting that normal metastasis-related processes may not be possible in these tumors. Moreover, at a critical size, tumor growth becomes dependent on the ability to secure the blood supply via the development of a vasculature [31]. This process is blocked by antiangiogenic therapies, which reduce angiogenesis and inhibit tumor growth and metastasis [32, 33]. Thus, the potential facilitation of metastasis during siANGPTL4 therapy should be considered.

ANGPTLs are orphan ligands, as they do not bind to either the angiopoietin receptor tyrosine kinase Tie2 or VEGFRs [7, 34]. Several studies have shown that ANGPTL4 modulates lipid metabolism by inhibiting the activity of lipoprotein lipase (LPL), an enzyme that hydrolyzes triglycerides (TGs) in lipoproteins, including chylomicrons, very low-density lipoproteins (VLDLs), fatty acids, and cholesterol [35]. Considering the potential effect of this angiopoietin-like protein on lipid metabolism, a possible relation of ANGPTL4 with obesity was explored. Kim et al. emphasized the key roles of ANGPTL4 in mediating energy metabolism in different murine models [36], and Robciuc et al. reported a positive association among ANGPTL4 expression, hormone-sensitive lipase expression in adipose tissue, and abhydrolase domain containing 5 (*ABHD5*) gene expression in an interesting study on monozygotic twins, supporting the hypothesis that ANGPTL4 plays a role in adipocyte lipolysis [37]. In addition, a previous study reported that protein kinase B (AKT/PKB) is associated with the regulation of glucose consumption and glycolysis in cancer cells that have undergone metabolic reprogramming [38]. Combining these results with the capability of ANGPTL4 to modulate the function of AKT/PKB in conferring anoikis resistance on tumor cells, it remains to be confirmed whether ANGPTL4 also alters cancer cell metabolism [39]. Although no alterations in the migration, invasion, and proliferation abilities of ANGPTL4 knockdown A2780 and CAOV3 ovarian cancer cells were observed in our in vitro experiments (Fig. 3), further studies to determine the impact that ANGPTL4 may have on ovarian cancer cell metabolism will be interesting.

There are discordant data about whether ANGPTL4 plays a proangiogenic or antiangiogenic role in tumors. Recent studies reported that ANGPTL4 promotes the secretion of diverse proangiogenic factors independent of VEGF. In this regard, many in vitro and in vivo experiments have confirmed dramatic elevation of ANGPTL4 in endothelial cells to express a deregulated herpesvirus-8 (HHV-8)-encoded G protein-coupled receptor (GPCR), which is considered a key factor in Kaposi’s sarcoma tumorigenesis [40]. Consistent with these findings, ANGPTL4 deletion has been related to a significant reduction in angiogenesis and vascular leakage in vitro and GPCR-regulated tumorigenesis in vivo [10, 41]. Moreover, a clinical study in uveal melanoma patients indicated that ANGPTL4 secretion is controlled by hypoxia-inducible factor-1 (HIF-1) and interactions with VEGF to enhance angiogenesis, implying the potential benefit of combined inhibition of ANGPTL4 and VEGF to improve the efficacy of antiangiogenic therapy [42]. Other studies have revealed that ANGPTL4 is an antiangiogenic agent that decreases the proliferation, tube formation, and chemotaxis of endothelial cells. Ito and colleagues implied the roles of ANGPTL4 in the mouse epidermis by using a neovascularization assay, suggesting that ANGPTL4 inhibits only VEGF-mediated neovascularization, whereas it cannot affect VEGF-independent angiogenesis [9]. Collectively, this evidence indicates that the proangiogenic and antiangiogenic roles of ANGPTL4 are tissue-specific and strongly dependent on the tumor properties. While we did not investigate the connection between ANGPTL4 and VEGF in neoangiogenesis, our data suggest the proangiogenic role of ANGPTL4 in epithelial ovarian cancer, in which it increases tumor neovascularization and supports tumor growth and metastasis. In addition, ANGPTL4 is highly expressed in A2780 and MISIIR-TAg-induced ovarian carcinoma cells and in CAOV3 and OVCAR3 ovarian adenocarcinoma cells (Fig. 1a–c, Additional file 1: Fig. S1a); however, the low to negligible levels of ANGPTL4 in other types of ovarian tumors limit these conclusions.

Taken together, our data suggest that specifically suppressing ANGPTL4 in ovarian tumors decreases vascular angiogenesis through dissociation of the VEGFR2/VE-cadherin/Src complex and phosphorylation of VEGFR2 at Y949 and therefore inhibits tumor growth and metastasis. With the intensifying investigation of antiangiogenic therapies, research targeting ANGPTL4 may offer a strategy with great potential in future drug development to reduce the tumor burden and to improve therapeutic efficacy in ovarian cancer.

## Supplementary Information

**Additional file 1**: **Fig. S1**. a Western blotting and statistical graphic for ANGPTL4 in ovarian tumors of WT, VEGFR2Y949F/Y949F (Y949F), MISIIR-TAg WT (TAg-WT), and MISIIR-TAg Y949F (TAg-Y949F) mice transfected with siANG or Scrambled siRNA control to testify the ANGPTL4 inhibition, n=3 independent experiments per group. NOTE: WT and Y949F groups in Fig.6 correspond to TAg-WT and TAg-Y949F mice. b qRT-PCR to verify the ANGPTL4 mRNA expression in ovarian tumors of transgenic mice, n=3 independent experiments per group. c Western blotting and VEGFR2 pY949 expression normalized to VEGFR2 or GAPDH in ovarian tumors of transfected transgenic mice to confirm the inactivation of VEGFR2 at Y949, n=3 independent experiments per group. d A representative image of reproductive tract of MISIIR-TAg mouse exhibiting bilateral ovarian tumors. Arrowheads indicate ovarian tumors. Scale bar: 0.5 cm. ***p < 0.001 with indicated groups. Data were presented as mean ± SEM. siANG, small interfering RNA targeting ANGPTL4. **Fig. S2**. No difference in tumor growth was observed in WT and VEGFR2 Y949F/Y949F mice with control vehicle treatment. a Experimental strategy. TAg-WT (WT) and TAg-VEGFR2Y949F/Y949F (Y949F) mice were constructed and pups were evaluated for sex, and tail tips were acquired from female mice for genotyping. On postnatal day (pnd) 28, mice were injected with control lentiviruses into their ovaries. On pnd 56, mice were sacrificed for further analysis. b Ovarian tumor volumes in WT-Ctl and Y949F-Ctl mice on pnd 56, n=9-10 mice per genotype. c Tumor diameter distribution (1-5 mm and >5 mm) on pnd 56, n=9-10 mice per genotype. d Immunofluorescent staining and statistical graphic of VE-cadherin in WT-Ctl and Y949F-Ctl tumors, n=4 tumors per genotype. Scale bar: 50 μm. e Western blotting for VE-cadherin and GAPDH in WT-Ctl and Y949F-Ctl tumors, n=4 tumors per genotype. f Western blotting for slug, snail, and GAPDH in WT-Ctl and Y949F-Ctl tumors, n=4 tumors per genotype. ns indicates no significance. Data were presented as mean ± SEM. **Table S1**. Primer sequences used for PCR and siRNA sequences.

## Data Availability

The datasets obtained and analyzed during the current study will be made available from the corresponding authors upon request.

## Abbreviations

ANGPTL4: Angiopoietin-Like 4
EdU: 5-Ethynyl-2′-deoxyuridine
EMT: Epithelial-mesenchymal transition
MISIIR: Mullerian inhibitory substance type II receptor
qRT-PCR: Quantitative reverse transcription PCR
RNAi: RNA interference
siRNA: Small interfering RNA
TGF-β2: Transforming growth factor-beta 2
VE-cadherin: Vascular endothelial-cadherin
VEGFR2: Vascular endothelial growth factor receptor 2
