# Supplementary material for: Deregulation of angiopoietin-like 4 slows ovarian cancer progression through vascular endothelial growth factor receptor 2 phosphorylation
Source: Cancer Cell Int. 2021 Mar 16;21:171. doi: 10.1186/s12935-021-01865-4 (PMC7968256; doi:10.1186/s12935-021-01865-4)
Supplement: Supplementary file 1 — Additional file 1: Fig. S1. a Western blotting and statistical graphic for ANGPTL4 in ovarian tumors of WT, VEGFR2Y949F/Y949F (Y949F), MISIIR-TAg WT (TAg-WT), and MISIIR-TAg Y949F (TAg-Y949F) mice transfected with siANG or Scrambled siRNA control to testify the ANGPTL4 inhibition, n=3 independent experiments per group. NOTE: WT and Y949F groups in Fig.6 correspond to TAg-WT and TAg-Y949F mice. b qRT-PCR to verify the ANGPTL4 mRNA expression in ovarian tumors of transgenic mice, n=3 independent experiments per group. c Western blotting and VEGFR2 pY949 expression normalized to VEGFR2 or GAPDH in ovarian tumors of transfected transgenic mice to confirm the inactivation of VEGFR2 at Y949, n=3 independent experiments per group. d A representative image of reproductive tract of MISIIR-TAg mouse exhibiting bilateral ovarian tumors. Arrowheads indicate ovarian tumors. Scale bar: 0.5 cm. ***p < 0.001 with indicated groups. Data were presented as mean ± SEM. siANG, small interfering RNA targeting ANGPTL4. Fig. S2. No difference in tumor growth was observed in WT and VEGFR2 Y949F/Y949F mice with control vehicle treatment. a Experimental strategy. TAg-WT (WT) and TAg-VEGFR2Y949F/Y949F (Y949F) mice were constructed and pups were evaluated for sex, and tail tips were acquired from female mice for genotyping. On postnatal day (pnd) 28, mice were injected with control lentiviruses into their ovaries. On pnd 56, mice were sacrificed for further analysis. b Ovarian tumor volumes in WT-Ctl and Y949F-Ctl mice on pnd 56, n=9-10 mice per genotype. c Tumor diameter distribution (1-5 mm and >5 mm) on pnd 56, n=9-10 mice per genotype. d Immunofluorescent staining and statistical graphic of VE-cadherin in WT-Ctl and Y949F-Ctl tumors, n=4 tumors per genotype. Scale bar: 50 μm. e Western blotting for VE-cadherin and GAPDH in WT-Ctl and Y949F-Ctl tumors, n=4 tumors per genotype. f Western blotting for slug, snail, and GAPDH in WT-Ctl and Y949F-Ctl tumors, n=4 tumors per genotype. ns indicates [file 12935_2021_1865_MOESM1_ESM.docx]

**Supplementary Materials and Methods**

*VEGFR2^Y949F/Y949F^* mouse model

Velocigene technology was used to operate the sequence exchange of amino acid Y949 for phenylalanine in the 5’ sequence corresponding to exon 21 flanking a LoxP-restricted Neomycin cassette. The exchange was introduced via homologous recombination into F1H4 embryonic stem cells of 129S6/C57BL/6 background. The cassette was removed by Cre recombinase treatment, leading to an 82 bp deletion in intron 21. The correct introduction of the mutation and the otherwise unaffected sequence of exon 21 and 22 was verified by nucleotide sequencing on both strands of DNA from tail tips clipped from *VEGFR2^Y949F/Y949F^* mice. Inactivation of VEGFR2 at Y949 in *VEGFR2^Y949F/Y949F^* mice was identified in Supplementary Fig. S1c.

MISIIR-TAg mouse model

The MISIIR-TAg transgene is a fusion construct containing the 5’ upstream regulatory sequences of the murine MISIIR gene upstream of the SV40-TAg (primer sequences were shown in Supplementary Table S1). Day-0.5 embryos of hybrid C57BL/6/B6C3F1 background mice were microinjected with the fusion genes, and embryos were implanted into oviducts of day-0.5 pseudopregnant C57BL/6 females. Transgenic offspring were backcrossed for 12 generations with C57BL/6 mice. Female MISIIR-TAg mice develop a high incidence of spontaneous bilateral epithelial ovarian carcinoma (See Supplementary Fig. S1d). Transgenic mice were identified by RT-PCR amplification of the MISIIR promoter from tail DNA.

**
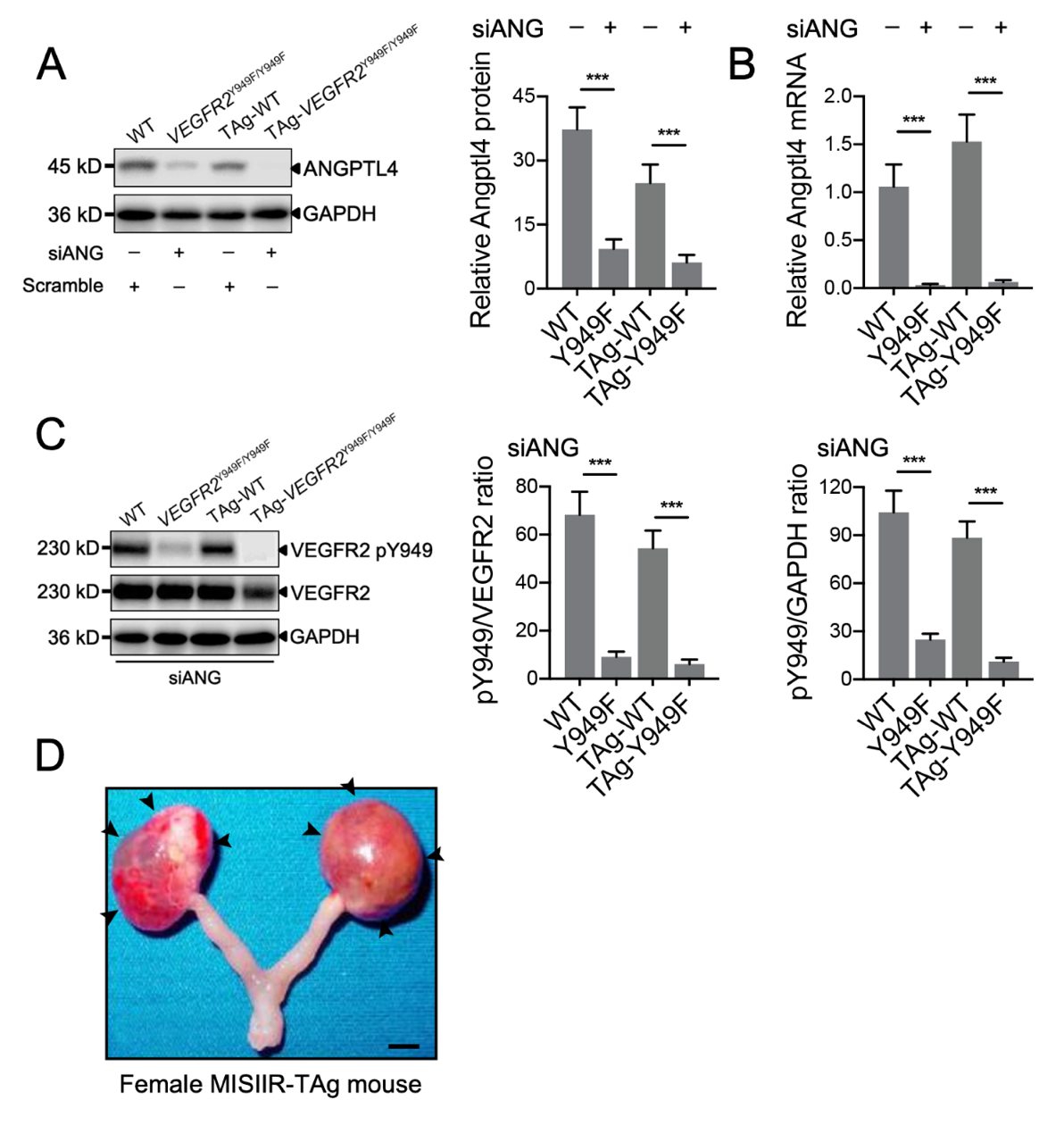
Supplementary Figures and Figure legend**

**Supplementary Fig. S1** **a** Western blotting and statistical graphic for ANGPTL4 in ovarian tumors of WT, VEGFR2Y949F/Y949F (Y949F), MISIIR-TAg WT (TAg-WT), and MISIIR-TAg Y949F (TAg-Y949F) mice transfected with siANG or Scrambled siRNA control to testify the ANGPTL4 inhibition, n=3 independent experiments per group. NOTE: WT and Y949F groups in Fig.6 correspond to TAg-WT and TAg-Y949F mice. **b** qRT-PCR to verify the ANGPTL4 mRNA expression in ovarian tumors of transgenic mice, n=3 independent experiments per group. **c** Western blotting and VEGFR2 pY949 expression normalized to VEGFR2 or GAPDH in ovarian tumors of transfected transgenic mice to confirm the inactivation of VEGFR2 at Y949, n=3 independent experiments per group. **d** A representative image of reproductive tract of MISIIR-TAg mouse exhibiting bilateral ovarian tumors. Arrowheads indicate ovarian tumors. Scale bar: 0.5 cm. ***p < 0.001 with indicated groups. Data were presented as mean ± SEM. siANG, small interfering RNA targeting ANGPTL4.


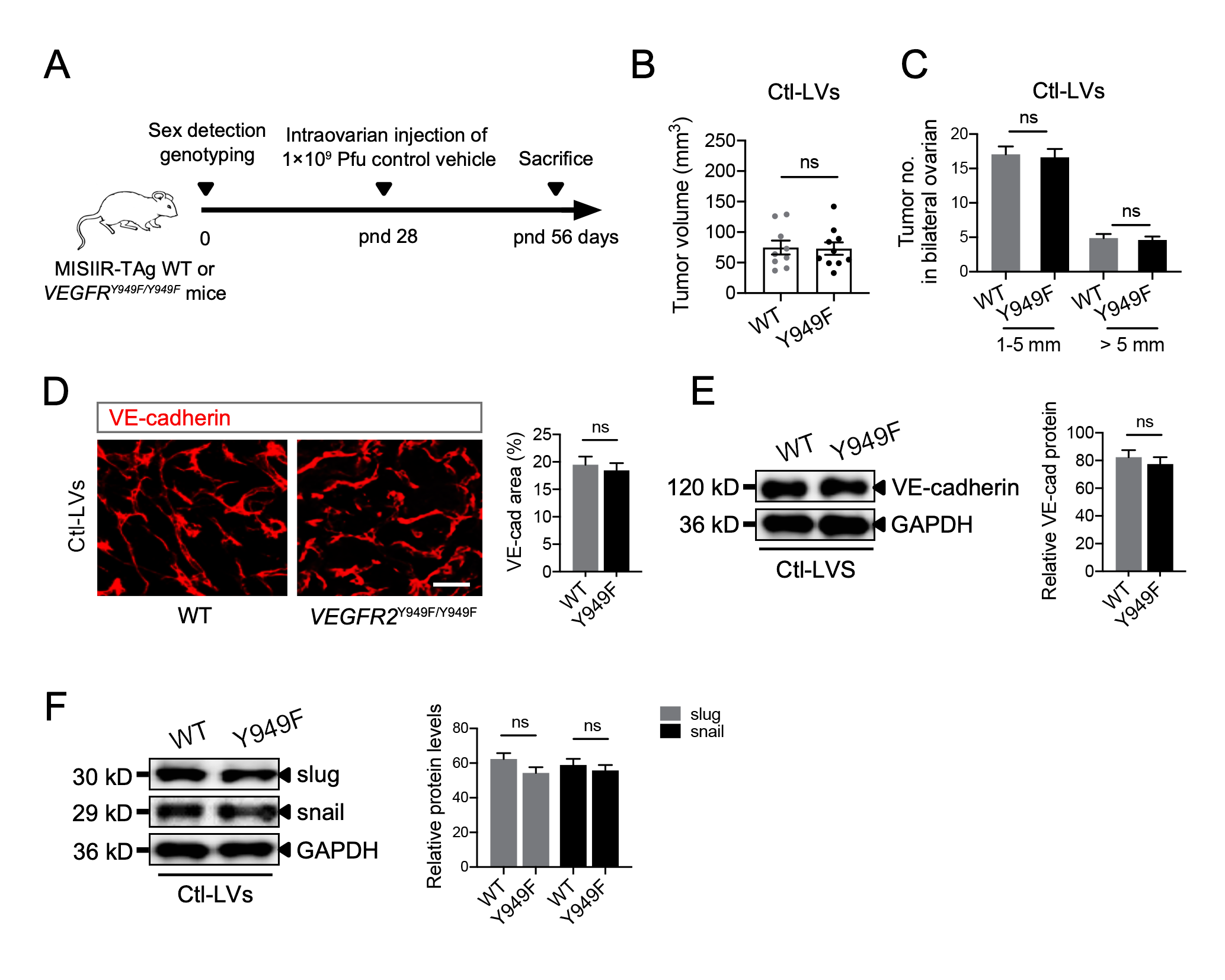


**Supplementary Fig. S2 No difference in tumor growth was observed in WT and *VEGFR2* *^Y949F/Y949F^* mice with control vehicle treatment.** **a** Experimental strategy. TAg-WT (WT) and TAg-*VEGFR2^Y949F/Y949F^* (Y949F) mice were constructed and pups were evaluated for sex, and tail tips were acquired from female mice for genotyping. On postnatal day (pnd) 28, mice were injected with control lentiviruses into their ovaries. On pnd 56, mice were sacrificed for further analysis. **b** Ovarian tumor volumes in WT-Ctl and Y949F-Ctl mice on pnd 56, n=9-10 mice per genotype. **c** Tumor diameter distribution (1-5 mm and >5 mm) on pnd 56, n=9-10 mice per genotype. **d** Immunofluorescent staining and statistical graphic of VE-cadherin in WT-Ctl and Y949F-Ctl tumors, n=4 tumors per genotype. Scale bar: 50 μm. **e** Western blotting for VE-cadherin and GAPDH in WT-Ctl and Y949F-Ctl tumors, n=4 tumors per genotype. **f** Western blotting for slug, snail, and GAPDH in WT-Ctl and Y949F-Ctl tumors, n=4 tumors per genotype. ns indicates no significance. Data were presented as mean ± SEM.

**Supplementary Table S1. Primer sequences used for PCR and siRNA sequences.**

| siRNA sequences | |
| --- | --- |
|  | Sequences (5' to 3') |
| ANGPTL4 siRNA | CTG CGA ATT CAG CAT CTG CAA |
| Scrambled siRNA | TGC CTT CAG ACT AGC AAT GAC |
| Primer sequences used for PCR | |
| Gene | Primer sequences (5' to 3') |
| MISIIR-F | CAG CCA GAA TGT GCT CAT TCG |
| MISIIR-R | GCT CAG TAT CTC CCA CAG TAG |
| ANGPTL4-F | GGC TCA GTG GAC TTC AAC CG |
| ANGPTL4-R | CCG TGA TGC TAT GCA CCT TCT |
| VE-cadherin-F | AAG GAC ACT GGC GAA AAC CT |
| VE-cadherin-R | ACG CAT TGA ACA ACC GAT GC |
| GAPDH-F | GGA GCG AGA CCC CAC TAA CAT |
| GAPDH-R | ACA TAC TCA GCA CCG GCC TC |
